# Construct validity of the Dining Environment Audit Protocol: a secondary data analysis of the Making Most of Mealtimes (M3) study

**DOI:** 10.1186/s12877-018-0708-4

**Published:** 2018-01-22

**Authors:** Sabrina Iuglio, Heather Keller, Habib Chaudhury, Susan E. Slaughter, Christina Lengyel, Jill Morrison, Veronique Boscart, Natalie Carrier

**Affiliations:** 1 University of Waterloo, Waterloo, Canada; 2 Schlegel-UW Research Institute for Aging, University of Waterloo, 200 University Ave W, Waterloo, On N2L 3G1 Canada; 3 Simon Fraser University, Vancouver, Canada; 4 University of Alberta, Edmonton, Canada; 5 University of Manitoba, Winnipeg, Canada; 6 Conestoga College; Schlegel-UW Research Institute for Aging, Waterloo, Canada; 7 University of Moncton, Moncton, Canada

## Abstract

**Background:**

Research has demonstrated the importance of physical environments at mealtimes for residents in long term care (LTC). However, a lack of a standardized measurement to assess physical dining environments has resulted in inconsistent research with potentially invalid and unreliable conclusions. The development of a standardized, construct valid instrument that assesses dining rooms is imperative to systematically examine physical environments in LTC. The purpose of this study was to determine the construct validity of the new Dining Environment Audit Protocol (DEAP) tool.

**Methods:**

Secondary data collected from the Making Most of Mealtimes (M3) study was used for this analysis. Data were collected in 32 long term care homes, which included 82 dining rooms and 639 residents. A variety of resident and dining room level constructs were compared to the summative scales found on the DEAP using Spearman correlations and Student t-tests. A regression analysis identified individual characteristics assessed with DEAP that were associated with the summative scales of homelikeness and functionality.

**Results:**

Regression analysis (*p* < 0.05) identified that the DEAP homelikeness scale was positively associated with a view of the garden/green space, presence of a clock and a posted menu. The functionality scale was positively associated with number of chairs and lighting, while negatively associated with furniture with rounded edges and clutter. Additionally, the functionality scale was positively associated (*p* < 0.05) with the Mealtime Scan physical scale (ρ = 0.52), the dining room Mealtime-Relational Care Checklist (M-RCC) (ρ = 0.25), the DEAP total score (ρ = 0.56), and the Mini Nutritional Assessment- Short Form (ρ = 0.26). Homelikeness was positively associated (*p* < 0.05) with the DEAP total score (ρ = 0.53), staff Person Directed Care score (ρ = 0.49) and the resident Cognitive Performance Scale (*t* = 2.56), while negatively associated with energy (ρ = −0.26) and protein intake (ρ = −0.24). The homelikeness and functionality scales were also associated with one another (ρ = 0.26).

**Conclusion:**

The construct validity of the DEAP was supported through significant correlations with a variety of measures that are theoretically related to the homelikeness and functionality of LTC dining rooms. This secondary analysis supports the use of the DEAP in future research to quantify the physical environment of LTC dining rooms.

Protocol registered with ClinicalTrials.gov ID: NCT02800291; Registered retrospectively June 7, 2016.

## Background

Resident malnutrition is a prevalent problem in long term care (LTC) homes that is both treatable and preventable [1–4]. Research has demonstrated that the physical environment has an important impact on the dining experience of LTC residents, supporting them to thrive in their environment by increasing social interaction [5], reducing agitation [6], increasing energy intake [7–11] and improving nutritional status [7]. Yet our understanding of the importance of the stable physical features of dining rooms is limited, as until recently, there was no face valid, reliable instrument that could be used to specifically assess these features. A newly developed tool, the Dining Environment Audit Protocol (DEAP) developed by Chaudhury et al. (2015), is based on environmental design features for dementia care [12]; the tool has been tested for inter-rater reliability [13]. DEAP is the first standardized environmental assessment specific to the physical aspects of the dining room and is based on the concept of competence-press and how the physical environment can support or produce barriers for psychosocial participation for residents [13]. A literature review identified seven therapeutic goals (e.g. orientation) of dining spaces in LTC [13]. These were translated into observable items that are typically static in a dining room that were included on DEAP [14]: general description of the physical space (e.g. number of tables and chairs); a drawing of the layout; and ratings on adequacy of lighting, presence of glare, length of pathways, clutter, capacity for staff supervision, use of restraints and potential for resident opinions on comfort of space (e.g. temperature, lighting) to be accommodated, and types of seating arrangements [13]. The two scales on DEAP summarize these aspects into functionality and homelikeness scores. Both scales range from 1 to 8 where a higher score indicates a greater degree of homelikeness or functionality [12, 13].

A key concept assessed by DEAP is homelikeness, based on features such as décor and adequate space in the dining room. Unfortunately, many LTC homes retain institutional features due to the adoption of the medical model [5] in their dining areas, such as inaccessible kitchens and lack of access to food or beverages between meals. More homelike dining rooms are associated with a higher levels of residents’ social interactions [5], higher calorie intake [11] and fewer periods of distress for those residents living with dementia [6]. Adjusting the lighting of the dining room also contributes to functionality as most older adults are sensitive to glare and also require increased lighting levels due to changes in their vision [15]. Adequate lighting is important in the dining room to accommodate these changes during mealtimes; sufficient lighting has been shown to be beneficial to nutrition outcomes [7], and quality of life [16].

Functionality is another key concept assessed by DEAP. Functionality includes safety and security, such as an appropriate dining room size with short pathways for food delivery that contain no clutter. Providing a safe route for residents to access the kitchen also enhances their feelings of autonomy and inclusion [14]. One study concluded that staff supervision in the dining area and the inclusion of noninstitutional features (e.g., no restraints) were associated with residents’ increased energy and fluid intake [10]. However, this study did not define what was observed in terms of “noninstitutional features” and only a simple counting of features was used to assess the degree of institutionalization of each environment [10].

DEAP also assesses the potential for social interactions by rating the space based on the availability of a variety of residents’ seating arrangements, such as a mix of large and small tables. Smaller and more homelike spaces can foster social interaction and prior research suggests the importance of social and homelike spaces for residents. One study invited six residents to have their meals in a small dining room and found that a more intimate environment, which included homelike décor and a less institutional atmosphere, promoted social interactions, satisfaction and ultimately enhanced quality of life and food intake [1]. However, these observations were subjectively determined and the features of the physical environment were not rated objectively. Being able to rate the static components of the environment is important to understanding their relevance with respect to food intake, as how individuals act or interact in such an environment cannot be disentangled at this point from these physical features. Providing residents with homelike dining features such as an open kitchen concept, adequate lighting, limited dining room clutter and homelike furniture and finishings have been found to support independence and autonomy, [14] and is related to higher food intake and enhanced overall quality of life [1, 9, 11, 17]. Yet, poor measurement of these features to date limits internal and external validity, and the interpretation of findings. DEAP has the potential to add to our understanding of how dining spaces can be configured to be more homelike, functional and support social interactions for residents. Use of a standardized tool with concepts supported by scientific evidence [1, 5–7, 9–11, 14, 16–18] will aid in the evaluation and comparison of different dining spaces and provide objective values for use in research.

The purpose of this study was to determine the construct validity of the DEAP tool, and specifically the functional and homelikeness summative scales. This was completed by: a) determining associations of individual DEAP variables/characteristics with these summative scales when adjusting for other variables included on the tool, and b) determining the association of these summative scales with a variety of theoretically important constructs including resident nutritional status, food intake, person-centred care and cognitive performance.

## Methods

Secondary data collected from the Making Most of Mealtimes (M3) study is used for this analysis. The M3 study is a multi-site, cross sectional study that collected data from 32 LTC homes in four Canadian provinces: Alberta, Manitoba, New Brunswick and Ontario [19]. Data were collected at the resident, dining room, and home levels. The purposes of M3 were to: 1) determine the food and fluid intake of residents in LTC and compare these to recommendations, and 2) identify the predictors of food and fluid intake. DEAP was specifically included to measure the physical mealtime environment as a potential determinant of food and fluid intake.

### Sample

Eight LTC homes were purposively recruited in each of the four provinces. Homes that were considered for inclusion had: 1) been operating for at least 6 months, 2) a minimum of 50 residents that met the resident eligibility criteria, and 3) agreed to participate in the data collection providing full cooperation for all procedures. For-profit and not-for-profit homes were recruited and homes with special characteristics were chosen to promote sample diversity (e.g., culturally based homes) [19]. Within each home, data were collected on one to four randomly selected care units; 82 dining rooms were assessed during data collection. Eligible residents were randomly sampled from the units that were selected, with twenty residents included from each home; these residents were representative of the study units [19]. The eligibility criteria for resident participation included: 1) residing on the units selected, 2) being over the age of 65, 3) requiring a minimum of 2 h each day of nursing care, 4) residing in the home for at least 1 month, and 4) they, or a substitute decision maker, provided informed consent to participate if the resident had cognitive impairment (i.e., Cognitive Performance Scale 3+). Resident exclusion criteria included: 1) residing in the home for less than 1 month, 2) medically unstable at the time of recruitment (e.g. recent hospital transition), 3) short term admission at the time of recruitment, 4) requiring tube feeding, 5) deemed by home staff to be at the end of life, and/or 6) having an advanced directive that excluded them from research. A total of 640 residents were recruited with a final sample of 639, as one participant withdrew consent. Eligible staff included nursing, recreation and/or dietary that were regular part-time or full-time employees. A minimum of 10 employees working on the selected units were recruited for data collection [19]. A site survey was completed by home management to describe the homes and units included in the sample (e.g. profit status, part of a continuum of care, beds (total and study units), age of home, renovations in unit in past 5 years).

### Measures

#### The DEAP tool

While the DEAP tool has not been tested for construct validity, this tool has demonstrated inter-observer reliability [13]. The homelikeness and functionality summary scales had good intraclass correlation coefficient values of 0.68 and 0.70, respectively [13]. The DEAP tool was used to assess the physical environment in each dining area by a trained research coordinator from each of the four provinces; it consists of primarily observational components as well as two questions asked of staff members. DEAP is designed to be an assessment of the physical space to compare and contrast physical features only, among dining spaces. Thus the observations are performed once at the beginning of data collection for each home, when the dining room was empty. Assessors were trained to walk throughout the dining space, observing the room/area from a variety of vantage points to complete the tool. Information was first recorded about the unit and the dining room, specifically: unit type (dementia care unit or general care unit). Numbers of tables chairs stools or chairs for staff and entry ways/exits were counted. By considering table/chair position for residents and vantage points for windows, percentage of residents with a clear view of the outside garden/green space was rated (≤24%, 25–49%, 50–74% 75%+; score 0 to 3). The following items were noted dichotomously as present (yes = 1) or absent (no= 0): use of adjustable tables; contrast between floor/table/dishes; rounded edges of furniture; presence of a posted menu; detergents/non-edibles secured; stove and other dangerous items secured; presence of a television and/or clock; dining room open between meals (i.e., door open, space not locked or closed off between meals); adjacent family kitchen with residential appliances, private family dining area; short distance from most bedrooms and visible from bedrooms; accessible washroom near dining room; accessible beverage services; and accessible main kitchen/servery (i.e., no barrier/door limiting resident access between meals). Data were also collected on the functionality of the space, including lighting intensity (0 = poor, 1 = reasonable, 2 = plenty), and glare (0 = strong, 1 = some, 2 = minimal). Safety and security information was assessed by categorizing the space on the size of the dining room, length of pathways for meal delivery, presence of obstacles/clutter, the ability of staff to view all residents and the use of restraints. Ratings for size, pathway and obstacles/clutter were: one (e.g. large institutional space >30 residents; long pathways for meal delivery >25 ft; several obstacles/ clutter), two (e.g. moderately large space [20–30 residents]; moderate length of pathways [15–25 ft]; some clutter/obstacles) and three (e.g. homelike space <20 residents; short pathways for meal delivery <15 ft; no clutter/obstacles) with a higher score indicating a more functional dining room. Based on the size of the dining space and layout of servery and tables, the space was rated on capacity for staff members to supervise residents (i.e., could they feasibly view all residents in the dining space or were there potential obstacles). Large space and/or awkward layout that would hinder staff observation of residents was given a score of 0, staff being able to easily view and access almost all residents was provided a score of 1 and staff being able to view all residents and get to them easily and quickly was give a score of 2. Social potential of the space was rated by the presence of a mixture of seating arrangements on a scale from zero to two, signifying one option (score = 0), 2 or 3 options (score = 1) and >3 options of seating arrangements (score = 2). Although most aspects of DEAP are observed by assessors, information on two questions is attained from staff. Assessors were trained to ask staff if residents’ opinions on the physical environment (e.g., light, noise, temperature; scored as yes = 1, no = 0, unsure = 9) were respected and acted on and if physical restraints were used in the dining room (yes = 1 and no = 0; unsure = 9). After completing DEAP, the assessor subjectively rated the overall space on two separate scales, homelikeness and functionality of the environment, with the scales ranging from 1(low) to 8 (high) [14]. For analysis only, researchers derived a total score by tallying individual variables/characteristics using the above item coding, resulting in a maximum DEAP score of 56, where a higher score indicates more physical features that are supportive of dining.

#### DEAP staff training and protocol

Research coordinators were trained to complete all M3 measures, including the DEAP, during 3-days of in-person training. Specific to the DEAP, researchers reviewed the tool with coordinators question by question, to clarify intent of items. Pictures of LTC physical dining rooms were used to demonstrate the physical qualities to be attended to when scoring. The research coordinators then observed four dining rooms to practice their assessments; results were compared and clarification provided where required to promote consistency among raters [19].

#### Theoretical constructs for comparison

Two standardized measures collected at the home and dining room level were used to assess construct validity. The 50 item staff-completed Person-Directed Care (PDC) questionnaire assesses staff’s perceptions with respect to personhood, comfort care, autonomy, knowing the person and support for relationships [20]. The staff PDC questionnaire has demonstrated face validity and conceptually distinct constructs (Cronbach’s alpha 0.86–0.91) [20]. In the M3 study, the staff PDC questionnaire was completed by 10–20 staff that provided care in the study units. The Mealtime Scan (MTS) is an observational tool that assesses the physical and psychosocial environments [21] while a meal is being consumed. Assessors observe an entire mealtime from beginning to end (on average 1 h), assessing various physical, social and care activities with respect to mealtimes as a meal is in process. As each meal is unique, dependent on time of day, who is present and the types of interactions that occur, multiple observations are required to assess the mealtime environment. This tool is face valid and is based on theoretical domains of physical, social, and relationship and person-centred practices in the dining room [21]. Embedded within the MTS is the Mealtime- Relational Care Checklist (M-RCC) that can be used on its own to assess relationship and person-centred care (R/PCC) practices and behaviours exhibited by staff with residents during mealtimes. The MTS includes three summary scales, on a scale from 1(low) to 8 (high), to assess the physical, social and person-centred environments. The MTS has been deemed an inter-rater reliable tool with good intraclass correlations (0.65–0.85) for the three summary scales and M-RCC [21]. The MTS was completed by the trained provincial coordinator and occasionally by the trained research assistants due to scheduling challenges with data collection. Assessments of the dining environment using the Mealtime Scan were completed 4–6 times in each unit’s dining room (*n* = 82) with observations at breakfast, lunch and dinner; the mean of scales was used in analyses. Individual items on MTS (e.g. number of residents, staff, residents:staff) were extracted to help describe the dining environments.

Resident level measures were also used to determine the DEAP’s construct validity. The interRAI Long Term Care Form is a standardized assessment tool used to gather information on health, cognitive and quality of life domains of LTC residents [22]. The trained provincial coordinators collected this data by interviewing staff members that were familiar with the resident’s current care and behaviour; interviewing was necessary as a) some provinces did not routinely use the interRAI instruments, b) to promote consistency among assessments, and c) as care needs and characteristics were desired to be concurrent with other data collected in M3 [19]. The items from the interRAI Long Term Care Form that were used to determine construct validity of DEAP were the Cognitive Performance Scale (CPS; maximum score of 6, with a higher score indicating a higher degree of cognitive impairment) and the Depression Rating Scale (DRS; maximum score of 33, with a higher score indicating increased presence of depressive symptoms) [22–24]. Malnutrition risk was measured using the Mini Nutritional Assessment- Short Form (MNA-SF), a valid and reliable instrument for assessing nutritional risk [25, 26]. Food intake for each resident was collected for three non-consecutive days, including one weekend day; assessments of food intake for an individual resident typically occurred during a 10-day period. All three meals for each day (nine meals in total) were observed and food intake determined by weighing main plate items before and after meal consumption. Side dishes and beverages were estimated considering the home’s portion sizes and capacity of serving dishes. Food and fluid intake between meals were estimated by researchers asking staff/resident/or family members what had been consumed between meals and estimating portion sizes. Food and fluid consumption after the evening meal was estimated and recorded by home staff. The detailed process for collecting food and fluid intake data is presented in the protocol paper [19]. LTC recipes were gathered and assembled in the nutrient analysis program Food Processor (version 10.14.1) and average energy and protein intake was estimated across the days of intake for each resident in this analysis.

### Statistical analysis

Two analyses were used to determine construct validity of the DEAP summative scales. First, a regression analysis determined those DEAP variables that predicted the homelikeness and functionality summative scales to demonstrate if and how these two constructs are similar and different. Bivariate analysis determined those DEAP variables more highly associated with these scales; those that had a *p*-value <0.25 were included in the initial multivariate model. Final multivariate models resulted from inclusion of these variables and backwards elimination using a p-value of <0.05 to determine the order for removal and retention of variables. The final model for each scale was achieved when all variables had a p-value of <0.05; potential interactions were also assessed. When the multivariate model for functionality was assessed, the DEAP variable “respecting and responding to resident’s opinions” was found to interact with the variable “residents are able to see the dining area from their bedroom”. Both of these variables were eliminated from the multivariate model, as the first variable had missing data for 12/82 dining rooms and concerns about accuracy of reporting by home staff on this question, while the second variable was not included due to low prevalence (3/82). The number of exits was also eliminated from the multivariate model due to the inability to differentiate between one exit and open concept dining rooms; further, this variable was also highly skewed. Collinearity tests were performed using the tolerance values and Cooks d to gather information on the existing relationships between each of the variables that remained in the final model. As tolerance values were >0.2 in all models, it was determined that multicollinearity was not present. Upon conducting Cooks d, outliers were detected and removed; however, this did not alter the interpretation of the model.

The second analysis to determine construct validity for the DEAP summary scales of homelikeness and functionality was based on their association with theoretically relevant resident and home/unit level measures. Descriptive statistics were computed for the staff PDC, MTS scales, M-RCC, MNA-SF, CPS, DRS and resident energy and protein intake. A Spearman rho correlation was computed for each instrument with the homelikeness and functionality scales, with *p* < 0.05 indicating statistical significance. To determine the association between CPS and the homelikeness and functionality scales, the CPS score was dichotomized into none to mild cognitive impairment (scores 0–2) and moderate to severe cognitive impairment (scores 3–6). Using a Student t-test, it was determined if homelikeness and functionality varied by cognitive status. All analyses were performed using SAS University (version 9.4).

## Results

Table 1 provides an overview of characteristics of residents and Table 2 of homes and units. Almost a third (31.1%) of the residents were male and the total resident sample had a mean age of 86.8 (SD 7.8) years. Over half (55.2%) of all residents were categorized as having moderate to severe cognitive impairment (i.e. CPS score 3+). The average score for the DRS was 2.3 (SD 2.92). Just over two-thirds of homes (68.8%) participating in this study were classified as not-for-profit, and the average home had been in operation for 31.2 (SD 16.3) years. The average number of beds on a study unit was 34.2 (SD 17.95). Of the dining rooms observed, 24 were in dementia care units and 58 were in general care units.

**Table 1:** Characteristics of residents participating in the M3 sample (*n* = 639)

Characteristic	% (n)/ Mean (SD)
Gender, male	31.1% (199)
Age (years)	86.8 (7.83)
# of diagnoses	5.4 (2.03)
# of medications	7.5 (7.0)
Moderate to Severe Dementia Status (CPS 3+)^a^	55.2% (353)
Depression rating scale^a^ (0–33)	2.3 (2.92)
Mini-Nutritional Assessment –SF (0–14)	10.6 (2.53)

^a^Assessed with InterRaiLTCF

**Table 2:** Home and unit characteristics in the M3 sample (*n* = 32 homes; *n* = 82 units)

Characteristic	% (n)
Not for profit home	68.8 (22)
Part of continuum of care	31.2 (10)
Renovations to unit in past 5 years^a^	21.2 (17)
Dementia care unit	29.3 (24)
	Mean (SD)
Home Age	31.20 (16.31)
Total # of beds in home	134.8 (58.02)
Number of beds on study unit	34.2 (17.95)
# of Residents in dining room during a meal	25.1 (13.81)
# of Staff in dining room during a meal	3.39 (2.23)
Ratio of residents/staff in dining room	7.7 (4.38)

^**a**^Missing 2/82

Table 3 provides the prevalence of physical features in the dining rooms as assessed by DEAP and the associations between these individual variables and the homelikeness and functionality summary scales. Across the 82 dining rooms, 58.5% of dining rooms provided 75% or more residents with a clear view of the garden/green space, 48.8% had plenty of lighting, 36.6% did not have obstacles/clutter, 58.5% had a good physical environment that supported supervision, 67.9% had contrast between the dish and table, 66.1% had a menu posted and 78.1% were open between meals. Alternatively, only 8.5% provided residents with multiple seating options, 59.8% did not have adjustable tables, 20.7% were a short distance from bedrooms, 34.2% had a private family dining area and 73.2% did not have an accessible main kitchen. Only 35.4% of dining rooms were considered to be homelike in terms of size and 23.2% were classified as institutional based on the size and seating capacity of the dining room. Finally, the average DEAP functionality score was 5.3 (SD 1.2) while the homelikeness score was 4.5 (SD 1.4). As these scales range from 1 to 8, these ratings indicated that the physical space of dining rooms was moderately homelike and functional. At a *p* < 0.05 level, four items were uniquely associated with homelikeness (view of garden/green space, dangerous items secured, distance from rooms; number of chairs) and four with functionality (lighting intensity, clutter, rounded edges; number of exits); four items were associated with both scales (respecting residents opinion on temperature or lighting; presence of posted menu; presence of a clock; dining room visually accessible from bedrooms). These associations for the most part were logical and conceptually made sense with respect to the constructs of homelikeness and functionality and demonstrate that the summary scales are derived from these individual items by assessors.

**Table 3:** Descriptive statistics and associations for each DEAP variable with DEAP homelikeness and functionality scores (*n* = 82 dining rooms)

		Homelikeness			Functionality		
Variable	Dining Room %(n)	Mean (SD)	β	*P*-value	Mean (SD)	β	*P*-value
% of residents with a clear view of the garden							
≤ 24%	10.98 (9)	3.56 (0.73)	–	<0.0001^d*^	4.56 (1.13)	–	0.11^d^
25–49%	4.88 (4)	2.5 (1.73)	−1.06		5.5 (0.58)	0.94	
50–74%	25.61 (21)	5.05 (1.20)	1.49		5.10 (0.94)	0.54	
75%+	58.54 (48)	4.63 (1.36)	1.07		5.5 (1.22)	0.94	
Lighting intensity							
Poor	3.66 (3)	6.00 (0)	–	0.17^d^	4.33 (0.58)	–	0.003^d*^
Reasonable	47.56 (39)	4.51 (1.40)	1.49		4.95 (1.02)	0.62	
Plenty	48.78 (40)	4.40 (1.43)	1.60		5.7 (1.16)	1.37	
Glare							
Strong	10.98 (9)	4.44 (1.01)	–	0.65	5.56 (1.01)	–	0.51
Some	65.85 (54)	4.61 (1.51)	0.17		5.33 (1.05)	−0.22	
Minimal	23.17 (19)	4.26 (1.28)	−0.18		5.05 (1.47)	−0.50	
Respecting/ responding to resident opinion on light, noise and temperature^a^							
No	40.00 (28)	4.00 (1.39)	–	0.01^d*^	4.90 (1.03)	–	0.01^d*^
Yes	60.00 (42)	4.86 (1.39)	0.86		5.55 (0.99)	0.66	
Size of dining room							
Institutional	23.17 (19)	4.21 (1.72)	–	0.35	5.42 (0.96)	–	0.23^d^
Moderate	41.46 (34)	4.77 (1.16)	0.55		5.47 (1.02)	0.05	
Homelike	35.37 (29)	4.41 (1.45)	0.20		5.0 (1.36)	−0.42	
Pathway length							
Long	18.29 (15)	4.27 (1.87)	–	0.56	5.4 (1.12)	–	0.78
Moderate	46.34 (38)	4.68 (1.19)	0.42		5.34 (1.05)	−0.06	
Short	35.37 (29)	4.41 (1.43)	0.15		5.17 (1.31)	−0.23	
Obstacles/ clutter							
None	36.59 (30)	4.57 (1.28)	–	0.95	6.03 (0.96)	–	<0.0001^d*^
Some	54.88 (45)	4.47 (1.52)	−0.10		5.04 (0.90)	−0.99	
Several	8.54 (7)	4.57 (1.40)	0.00		3.71 (1.11)	−2.32	
Physical environment supporting supervision							
Low	9.76 (8)	4.63 (1.30)	–	0.29	5.13 (1.36)	–	0.21^d^
Moderate	31.71 (26)	4.85 (1.38)	0.22		5.0 (0.89)	−0.13	
Good	58.54 (48)	4.31 (1.43)	−0.31		5.48 (1.22)	0.35	
Mix seating arrangements							
One	42.68 (35)	4.14 (1.56)	–	0.12^d^	5.37 (1.24)	–	0.74
Few	48.78 (40)	4.8 (1.24)	0.66		5.28 (1.11)	−0.10	
Multiple	8.54 (7)	4.71 (1.25)	0.57		5.0 (1.00)	−0.37	
Adjustable tables							
No	59.76 (49)	4.41 (1.50)	–	0.42	5.29 (1.21)	–	0.95
Yes	40.24 (33)	4.67 (1.27)	0.26		5.30 (1.07)	0.017	
Contrast between dish and table							
No	32.10 (26)	4.19 (1.33)	–	0.19^d^	5.08 (1.23)	–	0.22^d^
Yes	67.90 (55)	4.64 (1.43)	0.44		5.42 (1.10)	0.34	
Contrast between table and floor							
No	47.56 (39)	4.56 (1.27)	–	0.75	5.21 (1.15)	–	0.51
Yes	52.44 (43)	4.47 (1.53)	−0.10		5.37 (1.16)	0.17	
Rounded edges of furniture^b^							
No	45.00 (36)	4.67 (1.46)	–	0.59	5.67 (0.93)	–	0.02^d*^
Yes	55.00 (44)	4.5 (1.53)	−0.17		5.07 (1.21)	−0.60	
Posted menu							
No	32.93 (27)	3.81 (1.21)	–	0.001^d*^	4.93 (1.04)	–	0.04^d*^
Yes	67.07 (55)	4.85 (1.38)	1.04		5.47 (1.17)	0.55	
Detergent/ non-edibles secured							
No	4.88 (4)	5.50 (1.29)	–	0.15^d^	5.50 (1.29)	–	0.71
Yes	95.12 (78)	4.46 (1.40)	−1.04		5.28 (1.15)	−0.22	
Stove and other dangerous items secured							
No	12.20 (10)	5.6 (1.17)	–	0.01^d*^	5.8 (0.79)	–	0.14^d^
Yes	87.80 (72)	4.36 (1.38)	−1.24		5.22 (1.18)	−0.58	
Servery / pass through							
No	45.12 (37)	4.41 (1.48)	–	0.54	5.24 (1.23)	–	0.73
Yes	54.88 (45)	4.6 (1.36)	0.19		5.33 (1.09)	0.09	
Television							
No	68.29 (56)	4.39 (1.41)	–	0.26	5.29 (1.22)	–	0.94
Yes	31.71 (26)	4.77 (1.39)	0.38		5.31 (1.01)	0.02	
Clock							
No	15.85 (13)	3.62 (1.26)	–	0.01^d*^	4.69 (1.18)	–	0.04^d*^
Yes	84.15 (69)	4.68 (1.38)	1.07		5.41 (1.12)	0.71	
Dining room open between meals							
No	21.95 (18)	4.5 (1.34)	–	0.97	5.39 (0.98)	–	0.69
Yes	78.05 (64)	4.52 (1.44)	0.016		5.27 (1.20)	−0.12	
Adjacent family kitchen							
No	73.17 (60)	4.56 (1.38)	–	0.69	5.28 (1.21)	–	0.90
Yes	26.83 (22)	4.41 (1.50)	−0.14		5.32 (0.99)	0.03	
Short distance from most bedrooms							
No	79.27 (65)	4.71 (1.37)	–	0.01^d*^	5.31 (1.16)	–	0.82
Yes	20.73 (17)	3.76 (1.35)	−0.94		5.24 (1.15)	−0.07	
Private family dining area							
No	65.85 (54)	4.39 (1.45)	–	0.27	5.17 (1.21)	–	0.17^d^
Yes	34.15 (28)	4.75 (1.32)	0.36		5.54 (1.00)	0.36	
Accessible washrooms near dining room^b^							
No	46.25 (37)	4.32 (1.43)	–	0.41	5.16 (1.43)	–	0.28
Yes	53.75 (43)	4.58 (1.35)	0.26		5.44 (1.16)	0.28	
Dining rooms visually accessible from most bedrooms							
No	96.34 (79)	4.58 (1.37)	–	0.02^d*^	5.37 (1.10)	–	0.002^d*^
Yes	3.66(3)	2.67 (1.15)	−1.92		3.33 (0.58)	−2.03	
Accessible beverage service							
No	57.32 (47)	4.34 (1.54)	–	0.20^d^	5.13 (1.21)	–	0.14^d^
Yes	42.68 (35)	4.74 (1.20)	0.40		5.51 (1.04)	0.39	
Accessible main kitchen/ servery							
No	73.17 (60)	4.55 (1.41)	–	0.69	5.40 (1.11)	–	0.16^d^
Yes	26.83 (22)	4.41 (1.44)	−0.14		5.00 (1.23)	−0.40	
Use of restraints^c^							
No	53.42 (39)	4.59 (1.46)	–	0.28	5.56 (0.94)	–	0.06^d^
Yes	46.58 (34)	4.24 (1.28)	−0.35		5.06 (1.28)	−0.51	
Continuous Variables							
Variables	Mean (SD) {Tertile Range}	Correlation	β	*P*-Value	Correlation	β	*P*-Value
Ease of pathway total score	6.57 (1.59) {4,6,9}	0.01	0.02	0.81	0.17	1.10	0.30
# of tables	9.82 (6.11) {0,8,31}	0.07	0.02	0.50	0.01	11.02	0.29
# of stools	2.66 (3.03) {0,2,12}	0.12	0.04	0.42	0.05	1.14	0.94
# of chairs	13.68 (7.92) {0,12,38}	0.20	0.04	0.05^d*^	0.09	0.00	0.12^d^
# of exits	2.80 (1.44) {1,2, 9}	−0.11	−0.12	0.24	0.29	2.36	0.01^d*^

Abbreviations: *n* number of dining rooms, *SD* standard deviation

^a^*n* = 70 due to the removal of the “unknown” category

^b^*n* = 80 due to missing data

^c^*n* = 73 due to the removal of the “unknown” category

^d^Variable included in regression analysis as *p* < 0.25

*indicate a *p*-value of <0.05

Table 4 provides the multivariate model for homelikeness. All variables significantly associated (*p* < 0.05) with homelikeness were associated in the expected direction and presented characteristics of the dining environment that conceptually enhance homelikeness. A view of the garden/green space (β (25–49%) = −2.25, β (50–74%) = 0.81, β (75%+) = 0.32, *p* < 0.01), presence of a clock (β = 0.77, *p* = 0.01), a posted menu (β = 1.03, *p* = <0.01) and number of chairs (β = 0.04, *p* = 0.04) were all positively associated (*p* < 0.05) with this score when adjusted for other covariates. The adjusted R^2^ squared was 0.35, indicating that these variables explained a good portion of the variance in this homelikeness summary scale.

**Table 4:** Final Multivariate Model of DEAP Items Associated with Homelikeness

Variable Name	Parameter Estimate	F Value	*P*-Value
View of garden		8.14	<0.0001
25–49% vs ≤24%	−2.25		
50–74% vs ≤24%	0.81		
75% + vs ≤24%	0.32		
Clock	0.77	4.74	0.03
Posted menu	1.03	12.91	0.0006
Number of Chairs	0.04	4.34	0.04

Used backwards regression to determine final model using *p* < 0.05

Table 5 provides the multivariate model for functionality. Adequate lighting (β (reasonable) = 0.85, β (plenty) = 1.32, p = 0.01) and the presence of excess obstacles and clutter (β (some) = −0.92, β (several) = −2.06, p < 0.01) were positively and negatively associated with functionality respectively. The number of chairs in the dining room (β = 0.03, *p* = 0.03) was positively associated with functionality, while furniture with rounded edges (β = −0.42, p = 0.03) was negatively associated with functionality. The adjusted R^2^ was 0.42 indicating that these variables explained a good portion of the variance for functionality.

**Table 5:** Final Multivariate Model of DEAP Items Associated with Functionality

Variable Name	Parameter Estimate	F Value	*P*-Value
Number of chairs	0.03	5.1	0.03
Furniture with rounded edges	−0.42	4.76	0.03
Obstacles/clutter		18.27	<0.0001
Some vs. None	−0.92		
Several vs. None	−2.06		
Adequate lighting		4.93	0.01
Reasonable vs. Poor	0.85		
Plenty vs Poor	1.32		

Used backwards regression to determine final model using *p* < 0.05

Table 6 provides the descriptive statistics for each of the measures used to further determine construct validity and their association with the DEAP summary scales. The DEAP homelikeness scale was positively associated with the DEAP functionality scale (ρ = 0.26, *p* = 0.02), the staff PDC scale (ρ = 0.49, *p* = <0.0001), and the DEAP total score (ρ = 0.53, *p* < 0.0001). The homelikeness scale was negatively associated with resident energy (ρ = −0.26, *p* = 0.04), and protein intake (ρ = −0.24, *p* = 0.03) and CPS (t (634) = 2.56, *p* = 0.01). The functionality scale was positively associated with the dining room level M-RCC ratio (ρ = 0.25, *p* = 0.02); MTS physical rating (ρ = 0.52, *p* = <0.0001); resident nutritional status (ρ = 0.26, *p* = 0.02) and the DEAP total score (ρ = 0.56, *p* < 0.0001).

**Table 6:** Descriptive Statistics and Associations of DEAP Functionality and Homelikeness Scales with other Measures

		Functionality		Homelikeness		
Variable	Mean(SD) {Tertile Range}	Spearman Correlation	*P*-Value	Spearman Correlation	*P*-Value	
Homelikeness Scale (*n* = 82)	4.51(1.41) {1,5,7}	0.26	0.02^*^			
Functionality Scale (*n* = 82)	5.29(1.15) {2,5,7}			0.26	0.02^*^	
DEAP Total Score	33.12(4.50) {24, 33, 45}	0.56	<0.0001^*^	0.53	<0.0001^*^	
Dining Room Level M-RCC positive:negative ratio (*n* = 82)	1.76(0.64) {0.99, 1.54, 4.43}	0.25	0.02^*^	0.20	0.07	
MTS Person-Centred Summary Scale (*n* = 82)	5.47(0.77) {2.25, 5.5, 7.5}	0.14	0.22	0.20	0.07	
MTS Physical Summary Scale (*n* = 82)	5.57(0.86) {2.75, 5.75, 7.5}	0.52	<0.0001^*^	0.18	0.10	
MTS Social Summary Scale (*n* = 82)	5.03(0.9) {2.25, 5.17, 7.25}	0.12	0.27	0.11	0.32	
Staff PDC Percentage Score (*n* = 461)	61.54 (5.49) {51.01, 61.7, 71.75}	0.10	0.35	0.49	<0.0001^*^	
DRS (*n* = 634)	2.32(2.92) {0, 1, 14}	0.02	0.84	−0.09	0.44	
Resident Energy Intake (kcal/kg bw) (*n* = 628)	24.55 (7.94) {1.87, 23.68, 90.07}	0.02	0.88	−0.26	0.02^*^	
Resident Protein Intake (protein g/kg bw)(*n* = 628)	0.91(0.35) {0.10, 0.86, 3.90}	−0.05	0.65	−0.24	0.03^*^	
MNA-SF (*n* = 638)	10.63 (2.53) {0, 11, 14}	0.26	0.02^*^	0.13	0.24	
CPS Score (*n* = 634)	Mean Function-ality (SD)	T Value	*P*-Value	Mean Home-likeness (SD)	T Value	*P*-Value
None to Mild (0–2)	5.42(1.06)	1.55	0.12	4.75(1.31)	2.56	0.01^*^
Moderate to Severe (3+)	5.29(1.01)			4.46(1.46)		

Abbreviations: *kcal/kg bw* kilocalorie per kilogram body weight, *SD* standard deviation, *M-RCC* Mealtime Relational Care Checklist, *DRS* Depression Rating Scale, *MTS* Mealtime Scan, *CPS* Cognitive Performance Scale, *MNA-SF* Mini Nutritional Assessment- Short Form

*indicates a *p*-value < 0.05

## Discussion

To date, there is little and inconsistent research on characteristics promoting homelikeness of the physical environment of dining rooms in long-term care homes. The purpose of the DEAP instrument is to quantitatively assess dining rooms, specifically the degree of homelikeness and functionality. The purpose of this study was to demonstrate the construct validity of the DEAP summative scales using data from 639 residents in 82 dining rooms from 32 nursing homes in four Canadian provinces. The bivariate analysis demonstrates the large amount of overlap that exists between the characteristics that improve homelikeness and functionality (Table 3). Many of these characteristics are supported in the literature especially lighting, colour contrasts and accessible beverage services during meals [7, 9, 11, 27]. These findings suggest that homelikeness and functionality are closely related, which is supported by the significant positive correlation found between these scales (Table 6; ρ = 0.26, *p* = 0.02).

Homelikeness ratings appear to be associated with a view of the garden/green space, having a clock, a posted menu and number of chairs in the dining room. Prior research that utilized an environmental design intervention on a dementia care unit suggests that agitation is reduced with the presence of a clock and signage [28]. Few studies have examined the effects of having a menu posted; however, it is a recommended practice [29] and improves awareness and orientation for residents [14]. The number of chairs in the dining room was positively associated with the DEAP homelikeness score. The mean number of chairs (μ = 13.7 (SD = 7.9)) was relatively few when considering the average number of residents (*n* = 25) in dining rooms [19]; thus more chairs in this analysis does not necessarily mean larger dining rooms but that regular chairs for seating were being used rather than wheelchairs [30]. Physical features of a clock, posted menu, dining chairs and windows appear to improve homelikeness of dining rooms and should be considered key design features for LTC.

The DEAP homelikeness scale was also associated with other constructs that were intuitive, such as the staff PDC score. Chaudhury et al. (2016) found that changes to the physical environment increased quality personal support (through meeting resident physical and psychosocial needs) and enhanced teamwork among staff by making mealtimes more enjoyable [31]. Other person-centred scales (e.g., MTS, PCC and M-RCC) were not quite significantly associated with the homelikeness score (*p* = 0.07), but were in the anticipated direction. This may be due to the minimal variance on the homelikeness summary score across the 82 dining rooms. In contrast with prior literature [10, 31], the DEAP homelikeness scale was negatively associated with residents’ energy and protein intake and not associated with nutritional status. Homelikeness is hypothesized to improve food intake by improving quality of life [32]. It is known that energy and protein intake as previously identified in this sample [33], are strongly associated with resident level factors such as eating challenges and requiring eating assistance; this negative bivariate association with homelikeness scores was also confirmed in multivariate analyses for energy intake [33]. This suggests that physical features of homelikeness, as measured by the DEAP, are not sufficient to improve food intake in residents of LTC. Finally, it was not surprising to find the negative association between homelikeness and the resident CPS score. Dementia care units often promote safety of residents and as evidenced in this study (data not shown), items such as an unsecured stove, were rare in these dining rooms. While these findings suggest that the homelike summary scale of the DEAP has construct validity, the adjusted R^2^ for the homelikeness model suggests that some variance in this scale was unexplained by DEAP variables. This may indicate that this summary scale has a subjective component that may be greater than the functionality scale, where the adjusted R^2^ was slightly higher.

Wheelchairs in dining rooms can decrease space for movement of staff and residents; consistent with this contention, functionality as measured by the DEAP summary scale was positively associated with the number of chairs in the dining rooms. Transferring residents from wheelchairs to dining room chairs also promotes resident dignity and dining experience [33]. Furniture with rounded edges was negatively associated with functionality in the dining room which was a surprising finding, as furniture with rounded edges is believed to improve safety [14, 34]. Functionality ratings may have been reduced in dining rooms with rounded edged furniture due to more challenges arranging round tables or reduction in navigation space. As expected, adequate lighting and fewer obstacles and clutter were positively associated with functionality [35, 36]. Clutter creates challenges when navigating the dining room and reduces residents’ feelings of autonomy and control [14], while proper lighting can encourage residents to be more mobile [36] and reduces glare [37]. Adequate lighting has been used in conjunction with other environmental interventions to improve residents’ nutritional health, food and fluid intake [9, 11] and improve functional independence [7]. Adequate lighting, reducing clutter and obstacles, and using regular chairs should be considered to promote functionality of dining rooms in LTC.

With respect to other constructs, the DEAP functionality summative scale was associated as anticipated with other measures. Associations with person-centred care measures (e.g., M-RCC, staff PDC) was expected as more space in the dining room has been found to allow staff to sit with the residents they are assisting [31] and improving functionality may allow staff members to work together efficiently [6, 31]. The consistency of ratings between the MTS physical scale and the DEAP functionality scale confirms that these two measures are capturing physical aspects of dining rooms. Finally, the positive association between functionality scores and nutritional status as measured by MNA-SF suggests that there is something occurring between physical dining spaces and nutrition. It is worth considering if prior research which found associations between physical ‘homelike’ changes and food intake [11, 38, 39] were actually capturing functionality changes or psychosocial processes measured in this study with the Mealtime Scan. As identified in this study homelikeness and functionality are correlated and have overlapping features. Nutritional status is the ultimate outcome of energy and nutrient intake meeting the requirements of the resident. The lack of association between functionality, and energy and protein intake however suggests the need for further research to understand a potentially complex relationship. For example, persons with better nutritional status may be located in more functional dining rooms as a result of personal characteristics (e.g., less dementia, eating challenges) that could be associated with both the functionality of the dining area and their food intake. Further work disentangling the associations observed in this study among food intake, nutritional status and physical dining environments is needed and it is further recommended that DEAP be completed with the Mealtime Scan in future research to ensure that meal to meal variations in physical features that can impact food intake are also captured.

### Limitations

The DEAP was collected when the dining room was empty rather than during mealtimes. While characteristics of the physical environment may be altered during mealtimes, the majority of characteristics assessed by the DEAP are those that are not easily altered. DEAP provides an assessment of these features, but does not extend to how these features may lead to noise and interactions in a dining space. This analysis cannot be considered representative of all LTC homes especially as study homes were purposively sampled. All of the measures used for construct validation have limitations. For example, the completion of the interRAI LTCF instrument required a single staff member to be interviewed by the provincial coordinator, which could introduce bias for the CPS and DRS scales. The staff who completed the PDC may not have been representative of other staff in these study homes. A significant potential for measurement bias was the necessity of having four assessors complete DEAP in each of the study provinces. Despite training, DEAP ratings of homelikeness and functionality likely include a subjective component. It was not considered feasible to determine reliability among the assessors collecting DEAP in the M3 study, and this is noted limitation of this work. All of these measurement challenges could have weakened the identified associations between the DEAP summary scales and these construct measures. Further, some of the individual items (e.g. clutter) on DEAP have poorer reliability, as noted in a prior investigation [13]. This reliability may have influenced the regression models used to further understand the constructs of homelikeness and functionality. Two items specifically had missing data, as they are difficult to assess; these were the two questions asked of staff on restraint use and if resident preferences with respect to temperature and lighting were considered. It is recommended that these items be removed from DEAP. Finally, some relevant aspects of mealtimes were not assessed in this study (e.g. types of staff involved in dining room care).

## Conclusions

This study demonstrates the construct validity of a new tool, the Dining Environment Audit Protocol, which provides for the first time, a standardized objective assessment of dining spaces in long term care with respect to homelikeness and functionality. Bivariate and multivariate associations between physical characteristics assessed on DEAP and these two constructs of the physical space suggest key aspects (e.g., clock, dining chairs, decreased clutter, view of garden/green space) that can be used to promote homelikeness and functionality in LTC dining rooms. Initial construct validation with other measures at resident, staff and dining room levels indicate that the DEAP summary scales are assessing relevant physical aspects of the dining room. Results of this work suggest that the physical space is associated with food intake and nutritional status, yet further work is required to disentangle what appears to be a complex relationship. Finally, the M3 study has demonstrated that the DEAP is a relevant research tool when completed by trained researchers.

## Abbreviations

CPS: Cognitive Performance Scale
DEAP: Dining Environment Audit Protocol
DRS: Depression Rating Scale
LTC: Long term care
MNA-SF: Mini Nutritional Assessment- Short Form
M-RCC: Mealtime-Relational Care Checklist
MTS: Mealtime Scan
PCC: Person-centred care
PDC: Person-Directed Care
RCC: Relational centered care
